# Editorial: EEG/MEG based diagnosis for psychiatric disorders

**DOI:** 10.3389/fnhum.2022.1061176

**Published:** 2022-11-02

**Authors:** Junpeng Zhang, Jing Xiang, Lizhu Luo, Rui Shui

**Affiliations:** ^1^Department of Automation, College of Electrical Engineering, Sichuan University, Chengdu, China; ^2^Departments of Pediatrics and Neurology, Cincinnati Children's Hospital Medical Center, Cincinnati, OH, United States; ^3^School of Life Science and Technology, University of Electronic Science and Technology of China, Chengdu, China

**Keywords:** psychiatric disorders, magnetoencephalography (MEG), EEG, machine learning, artificial intelligence

For a long time, the diagnosis and evaluation of psychiatric disorders are mainly based on clinical symptoms and signs, but the understanding of the etiology and pathogenesis of these psychiatric disorders such as schizophrenia and depression is still not completely clear. At present, there is a lack of objective neurobiological markers that can be used in clinical routine work such as clinical diagnosis, curative effect evaluation and prognosis evaluation of psychiatric disorders. Therefore, it is of great clinical significance to find biomarkers to improve the diagnosis level and evaluate the curative effect. Electroencephalogram (EEG) is a non-invasive technique to record the potential activity of biological brain, through which researchers can analyze the mechanisms underlying psychiatric disorders. In addition, machine learning can be used to further verify the role of these electrophysiological indicators in clinical diagnosis and curative effect evaluation of psychiatric disorders. The goal of this Research Topic is to advance research on neurobiomarkers and EEG/MEG-based diagnostic methods for mental disorders. We hope to conduct in-depth research on the pathogenesis and diagnostic measures of mental diseases such as schizophrenia and depressive disorder through EEG/MEG, in order to improve the performance of artificial intelligence for mental diseases diagnosing. Under this research theme, the following is a brief overview of nine published articles, which, respectively, studied the diagnosis, treatment, and future research of psychiatric disorders by using EEG/MEG.

In this issue, some work are about the research of diagnosis methods of mental disorders. The paper titled “Machine Learning-Based Electroencephalographic Phenotypes of Schizophrenia and Major Depressive Disorder” by Jang et al. investigates brain phenotyping in patients with schizophrenia (SZ) and major depressive disorder (MDD) using EEG and conducted machine-learning-based classification of the two diseases, using these EEG components.

Although EEG microstates have been suggested as a potential endophenotype for schizophrenia, no clear dynamic pattern of microstates has been found. The paper titled “EEG Microstates and Its Relationship With Clinical Symptoms in Patients With Schizophrenia” by Sun et al. demonstrates that patients with schizophrenia have abnormal EEG microstates, especially the microstate class C, through the method of grouping patients into subgroups according to the level of positive and negative symptoms.

Little research has explored EEG differences between adolescents with major depressive disorder (MDD) and healthy controls, particularly EEG microstates differences. The paper titled “Abnormalities in Electroencephalographic Microstates Among Adolescents With First Episode Major Depressive Disorder” by He et al. is the first study to explore the dynamic activity of resting-state large-scale brain networks among adolescents with MDD indicating that adolescents with MDD show EEG alterations in temporal scale of subsecond across the whole brain.

The paper titled “Detection of Schizophrenia Cases From Healthy Controls With Combination of Neurocognitive and Electrophysiological Features” by Tian et al. develops a comprehensive machine learning pipeline based on neurocognitive (contains seven specific areas of cognition) and electrophysiological [PPI, EEG power spectrum, detrended fluctuation analysis, and fractal dimension (FD)] features by using logistics, random forest, and extreme gradient boosting (XGBoost) algorithms and evaluated their classification capabilities separately.

Deep learning techniques have been applied to electroencephalogram (EEG) signals, with promising applications in the field of psychiatry. The paper titled “From Sound Perception to Automatic Detection of Schizophrenia: An EEG-Based Deep Learning Approach” by Barros et al. researches the altered patterns in electrical brain activity during auditory processing and their potential to discriminate schizophrenia and healthy subjects. Their results show the potential of deep learning methods in the study of impaired auditory processing in schizophrenia with implications for diagnosis.

Results of more recent studies have suggested that ASD is a dysfunction of coordination over widely distributed brain regions. To meet this challenge, the paper titled “Atypical Resting State Functional Neural Network in Children With Autism Spectrum Disorder: Graph Theory Approach” by Soma et al. examines the resting-state MEG-derived functional network in children with and without ASD using graph theory and demonstrates a difference between children with and without ASD in MEG-derived resting-state functional brain networks. Their study indicates that combining graph theory and MEG might be a promising approach to establish a biological marker for ASD.

Secondly, it is about exploring the treatment methods of these diseases. Transcranial direct current stimulation (tDCS) is an emerging therapeutic tool for treating posttraumatic stress disorder (PTSD). The paper titled “Predictions of tDCS treatment response in PTSD patients using EEG based classification” by Kim et al. investigates tDCS treatment responsiveness in patients with PTSD using EEG spectral power and machine learning-based prediction methods. These results can provide important information and provide meaningful methods for early identification of patients who may be clinically affected by tDCS treatment, thus reducing the cost and time spent by these patients in the treatment process. Their findings provide information for future research directions, and if confirmed, it is expected that they will eventually provide information for medical guidelines.

The paper titled “Repetitive Transcranial Magnetic Stimulation Modulates Frontal and Temporal Time-Varying EEG Network in Generalized Anxiety Disorder: A Pilot Study” by Song et al. investigates the effect of low-frequency rTMS targeting the right DLPFC on clinical symptoms and TMS-evoked time-varying brain network connectivity in patients with GAD. Their study demonstrates that rTMS does have potential as an effective augmentative treatment in GAD.

One of the work is about the analysis of the development status and trends of these research fields. The paper titled “Bibliometric Analysis of Quantitative Electroencephalogram Research in Neuropsychiatric Disorders From 2000 to 2020” by Yao et al. integrates bibliometric information on the current status, the knowledge base, and future directions of QEEG studies in neuropsychiatric disorders from a macroscopic perspective. It suggests that in the past 20 years, QEEG has been used to reveal the pathological mechanism of various neuropsychiatric diseases, to assist clinical diagnosis and to promote the selection of effective treatment methods. Besides, future studies should focus on cross-validation of promising QEEG biomarkers, development of noval biomarkers, and extraction of biomarkers by machine learning.

The work in this special issue may fall into three categories of research: (1) methods designed for diagnosis of psychiatric disorders; (2) methods for the treatment of psychiatric disorders; (3) trends in this field. AI techniques, including machine learning and deep learning, have been widely applied to improve diagnosis performance and treatment effectiveness. This issue will facilitate to deepen the understanding of the underlying mechanisms of psychiatric disorders from the aspects of neuroelectrophysiology and neuromagnetic-physiology with AI assistance, to improve the accuracy and convenience of treatment and also to inspire the development of better diagnostics and treatments.

## Author contributions

JZ and RS wrote the draft manuscript. JX and LL edited and improved it. All authors contributed to the article and approved the submitted version.

## Funding

This work was funded by Key Project of National Science of China (No. 12126606) and Sichuan Science and Technology Agency Project (No. 21ZDYF3607).

## Conflict of interest

The authors declare that the research was conducted in the absence of any commercial or financial relationships that could be construed as a potential conflict of interest.

## Publisher's note

All claims expressed in this article are solely those of the authors and do not necessarily represent those of their affiliated organizations, or those of the publisher, the editors and the reviewers. Any product that may be evaluated in this article, or claim that may be made by its manufacturer, is not guaranteed or endorsed by the publisher.

